# Body weight trajectories from midlife are associated with cognitive decline in advanced age

**DOI:** 10.1038/s41598-025-08725-5

**Published:** 2025-07-06

**Authors:** Chiara Ceolin, Federica Prinelli, Caterina Trevisan, Adele Ravelli, Silvia Conti, Lorraine Brennan, Lisette C. P. G. M. de Groot, Claire T. McEvoy, Stefania Maggi, Giuseppe Sergi, Marianna Noale, the PROMED-COG Consortium, Caterina Trevisan, Silvia Conti, Lorraine Brennan, Lisette C. P. G. M. de Groot, Claire T. McEvoy, Stefania Maggi, Giuseppe Sergi, Marianna Noale, Chris Cardwell, Bernadette McGuinness, Michelle C. McKinley, Jayne V. Woodside, Dorothee Volkert

**Affiliations:** 1 Geriatric Unit, Department of Medicine, University of Padova (UNIPD), Via Giustiniani 2, 35128 Padova, Italy; 2 Institute of Biomedical Technologies, National Research Council (CNR), Via Fratelli Cervi 93, 20054 Segrate, MI Italy; 3 Department of Medical Sciences, University of Ferrara, Ferrara, Italy; 4 School of Agriculture and Food Science, Institute of Food and Health and Conway Institute, University College Dublin, Belfield, Dublin, Ireland; 5 Division of Human Nutrition, Wageningen University, Wageningen, The Netherlands; 6 Centre for Public Health, Queen’s University Belfast, Belfast, Northern Ireland UK; 7 The Global Brain Institute, Trinity College Dublin, Dublin, Ireland; 8 University of California San Francisco, San Francisco, USA; 9 Neuroscience Institute, Aging Branch, National Research Council (CNR), Viale Giuseppe Colombo 3, 35121 Padova, Italy; 10 Institute for Biomedicine of Aging, Friedrich-Alexandra University of Erlangen-Nümberg, Erlangen, Germany; 11 Aging Research Center, Karolinska Institutet, Stockholm, Sweden

**Keywords:** Pooled cohorts, Trajectories analysis, Body weight, Cognitive decline, Dementia, Geriatrics, Risk factors, Predictive markers, Neurological disorders, Nutrition disorders

## Abstract

Fluctuations in body weight may impact cognitive decline, but current evidence is inconclusive. The aim of this study is to investigate associations between body weight trajectories from midlife to later life and cognitive decline. This retrospective study analyzed harmonized data from two population-based longitudinal studies, the Progetto Veneto Anziani and the Italian Longitudinal Study of Aging, encompassing baseline and two follow-up assessments over 9 years. Weight changes were recorded from baseline to the last available follow-up or from 50 years (self-reported data) to the last available follow-up. Cognitive function was assessed using the Mini-Mental State Examination (MMSE), and cognitive decline was defined as experiencing a MMSE change from baseline to the follow-up within the lowest quartile of the change distribution in the total sample. In a sample of 3852 individuals (46% females, age 65–96 years at baseline), we investigated the impact of weight change on cognitive decline with two sets of analyses. First, using weight measurements obtained during old age, growth mixture modelling identified three weight trajectories: decreasing, stable, and increasing. None of these trajectories was significantly associated with cognitive decline. Second, we considered weight at age 50 as the baseline assessment to capture weight changes from midlife. Among the three trajectories detected (increasing, stable, and decreasing), the decreasing trajectory was significantly associated with a higher likelihood of cognitive decline in males (HR 1.44, 95% CI 1.06–1.94) and females (HR = 1.37, 95%CI 1.23–1.67), whereas the increasing trajectory was associated with cognitive decline only in females (HR = 1.33, 95%CI 1.01–1.76). These results suggest that changes in body weight from middle to older age are associated with cognitive decline in advanced age. Since body weight is influenced by multiple factors, a broader assessment of health—including metabolic, vascular, behavioral, and social dimensions—should be considered in both research and clinical settings.

## Introduction

Aging is characterized by inevitable changes in body composition with an overall tendency to decrease body weight after age 60^1^, especially concerning bone and lean mass^2^. Although the contribution of fat mass to overall weight loss may be relatively small, its distribution varies with advancing age, with increased abdominal fat and ectopic infiltration^3,4^, accompanied by a marked reduction in subcutaneous fat mass^4^.

Recently, attention has been devoted to exploring the potential link between cognitive decline and fluctuations in body weight, given the burden dementia poses to the healthy aging of older individuals. Dementia, affecting over 50 million people worldwide, entails a gradual erosion of cognitive and functional abilities, accompanied by a spectrum of behavioral symptoms that unfold over the disease’s progression^5,6^. From early to advanced stages, cognitive impairments associated with dementia can alter meal planning and awareness of dietary needs and lead to behavioral disruptions that decrease caloric intake, resulting in weight loss^7^.

However, the relationship between dementia and weight loss is complex and bidirectional, and weight variations have also been shown to precede a diagnosis of dementia^8^. This is because body weight is not merely a marker of nutritional status, but may also reflect the presence of chronic conditions potentially influencing cognitive decline. Indeed, both excess weight due to unhealthy dietary habits (e.g. obesity-promoting Western diet^9–11^) and underweight with associated vitamin B and D deficiencies may predict steeper cognitive decline in older adults^12^. However, several other factors beyond nutritional status may influence body weight changes and cognitive function in advanced age, including multimorbidity, frailty, socioeconomic conditions, and physical activity levels. For instance, the coexistence of multiple chronic diseases may exacerbate the aging-related mild inflammatory status^13^ and affect muscle quantity and quality, physical capacity and, ultimately, cognitive function. Frailty, a syndrome characterized by diminished resilience, is another key factor. Indeed, previous studies showed that body weight changes (especially weight loss) predicted frailty development^14^, and that, in turn, the presence of frailty was associated with an almost twofold higher risk of cognitive disorders^15^. Social determinants are other crucial factors influencing both weight changes and cognitive decline in older age. Lower educational attainment, prior less cognitively demanding occupations, and poor housing conditions have been associated with a higher prevalence of undernutrition or excess weight conditions, often co-occurring with frailty^14^, as well as with a higher risk of cognitive disorders^15^. Finally, physical activity plays a pivotal role in regulating body composition by preserving muscle mass and counteracting fat accumulation and the deleterious effects of inflammaging^16^. However, the frequent decline in physical activity with age—ranging from 40 to 80%—can further predispose older adults to metabolic disorders and exacerbate body composition changes and cognitive decline^16^. In summary, a comprehensive understanding of cognitive health in older adults requires an integrated approach that considers all the factors potentially influencing body weight changes, not only nutritional status.

Nevertheless, the intricate relationship between weight fluctuations and cognitive disorders remains incompletely understood. While some studies suggest that higher weight or body mass index (BMI) at age 50 correlates with increased dementia risk, others indicate an inverse association in people aged 65 or older^5,17–21^. In particular, studies in older people found a U-shaped relationship, with underweight, overweight, or obese individuals more likely to develop dementia compared to those of normal weight^22,23^. Of note, studies addressing this issue have relatively short follow-ups^24^. A meta-analysis of 23 cohort studies found that participants who experienced a weight loss of 0.5% or more per year had a 28% higher risk of developing dementia. However, most of these studies had only a few years of follow-up and were all under 10 years^24,25^. Regarding cognitive decline, a Korean study reported that adults affected by obesity, particularly females, experienced a slower decline in cognitive function compared to those with normal weight^26^, while others underlined that excess weight may be more detrimental to cognitive health in midlife than in later life^27^.

In light of these results, we aimed to investigate whether body weight trajectories during adulthood and later life are associated with cognitive decline while also considering potential sex differences. We hypothesize that changes in body weight within individuals could offer valuable insights into the risk of cognitive decline, especially when these changes are tracked from middle age onward.

## Materials and methods

### Original studies

This study was performed as part of the project “PROtein enriched MEDiterranean diet to combat undernutrition and promote healthy neuroCOGnitive ageing” project (PROMED-COG), funded by the European Horizon 2020 Joint Programming Initiative “A Healthy Diet for a Healthy Life” (JPI-HDHL) and the ERA-NET Cofund ERA-HDHL, under the PREVNUT call for the promotion of strategies preventing undernutrition in older age. The specific goal of PROMED-COG is to assess the impact of undernutrition on neurocognitive ageing to develop interventions promoting healthy nutrition and cognitive ageing through collaborative research across Europe. Details on the project have been previously published^28^.

The first PROMED-COG activities aimed at investigating the impact of poor nutritional status on cognitive decline in advanced age. For this purpose, we considered a pooled cohort derived from three population-based studies, whose data were retrospectively harmonized. For this study, two of those population-based studies were included:

- The Italian Longitudinal Study of Aging (ILSA) is a longitudinal study that included 5632 individuals aged 65–84 years who were randomly selected and assessed in 1992–1993 (baseline). Participants were identified from the demographic lists of eight municipalities in Northern, Central, and Southern Italian regions. Follow-up evaluations were performed in 1995–1996 and 2000–2001. Baseline assessment consisted of two phases: the first phase included a personal interview about socio-demographic information, chronic conditions, risk factors, lifestyle characteristics, laboratory tests, a physical examination, and validated scales and questionnaires; the second phase was performed by specialists and involved only participants who screened positive to the first phase with the aim of clinically confirm diagnosis of cardiovascular, diabetes and neurological conditions^29,30^.
- The Progetto Veneto Anziani (Pro.V.A.^31^) is a longitudinal study involving 3099 adults aged ≥ 65 years living in Northern Italy, identified through an age- and sex-stratified random sampling procedure. The baseline assessment was performed in 1995–1997, and two active follow-ups were done in 1999–2000 and 2002–2004. Trained physicians and nurses assessed the participants at the research centers or home (for those who were unable to come to the center) through personal interviews and the administration of scales, questionnaires, and tests to collect data on socio-demographics, cognitive function, dietary habits, chronic diseases, and functional status. Physical examination and laboratory tests were also performed and reviewed by physicians to evaluate the presence of geriatric syndromes and chronic conditions.

The Pro.V.A. and ILSA study protocols were conducted in line with the guidelines of the Declaration of Helsinki, and were approved by local Ethics Committees (for the ILSA study, by the institutional review board of the eight participating municipalities; for the Pro.V.A. study, Ethical Committees of the University of Padova and the nrs. 15 and 18 Local Health Units of the Veneto Region). Written informed consent was obtained from all participants who took part in the studies.

### Data harmonization

Retrospective data harmonization was performed according to current recommendations^32^. For this study, we considered the information described below.

#### Main exposure

Body weight and height were measured during the physical examination with individuals wearing light indoor clothing and no shoes. Weight changes from baseline to the last available follow-up or from age 50 (self-reported data) to the last available follow-up were the main exposures of interest. In particular, we considered the percent weight change from the previous measurement in order to account for interindividual differences in baseline weight, providing a standardized metric across participants with varying body sizes.

#### Main outcomes

Cognitive function was assessed with the Mini-Mental State Examination^33^ at baseline and follow-ups. Cognitive decline at follow-up was operationalized as a dichotomous outcome, defined as an MMSE change within the lowest quartile (25%) of change distribution in the total sample.

#### Other variables

Socio-demographic data included participants’ sex, age, civil status, educational level, and work done most of the time (categorized into blue collars, white collars, housewives, Eurofound 2005). A composite measure of socioeconomic status (SES) was derived by attributing different scores to educational level (primary school or middle school: 1 point; high school or university or more: 2 points) and previous occupation (blue collar or housewife: score 1 point; white collar: score 2 points), obtaining a total score of 1 or 2 (low SES), 3 (medium SES), or 4 (high SES)^34,35^.

Regarding lifestyle characteristics, participants were classified as never smokers, former smokers, or current smokers. For alcohol consumption, heavy drinking was defined as exceeding 7 units per week for females and 14 units per week for males.

Among health-related factors, we evaluated the presence of arterial hypertension, diabetes mellitus, hyperlipidemia, cardiovascular diseases (CVD, defined as at least one among angina, ischaemic heart disease, arrhythmia, peripheral artery disease, or stroke), depressive symptoms, and limited mobility.

Nutritional status at baseline was evaluated based on the last Global Leadership Initiative on Malnutrition (GLIM) recommendations, defining the presence of undernutrition as the coexistence of at least one phenotypic and one etiologic criterion^36^. Among GLIM phenotypic criteria, the following data were collected: body weight, body mass index (BMI), mid-arm circumference^37^, and weight loss in the last year. Among GLIM etiologic criteria, we considered the presence of conditions associated with reduced food assimilation (bowel or stomach diseases, liver or gallbladder diseases) or inflammation (chronic bronchitis or emphysema or asthma, cancer, bone or joint diseases, heart failure), as well as blood tests indicating poor nutritional status or high inflammation (albumin < median values by age and sex; fibrinogen, white blood cell count ≥ median values by age and sex).

### Statistical analysis

Characteristics of the study participants were described as means ± standard deviation (SD), or median and interquartile range (IQR) for quantitative variables, and as counts and percentages for qualitative variables. These characteristics were compared using generalized linear models after testing for homoschedasticity (Levene test), Wilcoxon rank-sum test, Chi-square test, or Fisher exact test, as appropriate.

To identify distinct body weight change patterns over time, group-based trajectory modeling using SAS Proc Traj procedure was applied^38^. This method assumes that the population is composed of distinct latent groups, each following a different trajectory of weight change over time. Models were stratified by sex, and estimated trajectories based on percent weight change using follow-up wave (time of measurement) as the time variable. Several models were defined, ranging from 1 to 5-group trajectory models, with polynomials of different degrees for each group. The optimal number and shape of trajectories were identified by evaluating the Bayesian Information Criterion (BIC) as the difference between less and more complex models (2ΔBIC > 10), aiming for models where all groups comprised at least 5% of the sample and had posterior probability > 0.7 (indicating adequate internal reliability). The same procedure was applied to identify weight trajectories from age 50 to the last follow-up. Each participant was assigned to the trajectory group with the highest posterior probability. Figures displaying trajectory groups show the mean percentage weight change over time for each group, plotted against follow-up waves or age.

The associations between weight trajectories and cognitive decline were tested with Cox proportional hazard regression models stratified by sex.

Models were adjusted for the following covariates selected based on theoretical and clinical knowledge supporting their possible influence on cognitive performance^39^: age, educational level, lifetime main occupation, marital status, smoking habits, alcohol consumption, BMI, diabetes, hyperlipidemia, cardiovascular diseases, depression, limited mobility, and MMSE baseline scores. While we recognize that some of these variables may also act as potential mediators (e.g., depression, mobility), they were included to account for shared risk factors and provide conservative estimates. The proportional hazard assumption was verified considering Schoenfeld’s residuals of the covariates; interactions between variables were tested. Missing values were not imputed in primary analysis. Sensitivity analyses were performed including an alternative definition of cognitive decline based on Minimum Clinically Important Difference for MMSE (3 points, as suggested by Andrews et al.^40^) and after multiple imputation of missing data based on the fully conditional specification method (FCS) with 10 imputed data sets created and then combined using Proc MI Analyze. Additional sensitivity analyses evaluated group-based trajectories adjusting for age and sex, and then testing the interaction between sex and defined trajectories in relation to cognitive decline outcomes. For trajectories from age 50 to the follow-ups, further sensitivity analyses considered age instead of follow-up years as the time scale.

Two-tailed p-values < 0.05 were considered statistically significant. Analyses were performed using SAS statistical package, release 9.4 (SAS Institute Inc., Cary, NC).

## Results

From the total pooled sample of 8731, individuals living in institutions or nursing homes (n = 175), with incomplete baseline data (n = 1089) or missing information on the variables considered for the analyses (n = 3615) were excluded, resulting in a final analytical sample of 3,852 individuals (46% female) aged between 65 and 96 years at the baseline (Supplementary Fig. 1). Participants included in the analyses were significantly younger, more likely to be females, less educated, and had lower prevalence of diabetes, cardiovascular diseases (CVD), depressive symptoms, and walking disability compared with those excluded (Supplementary Table 1).

**Fig. 1:**
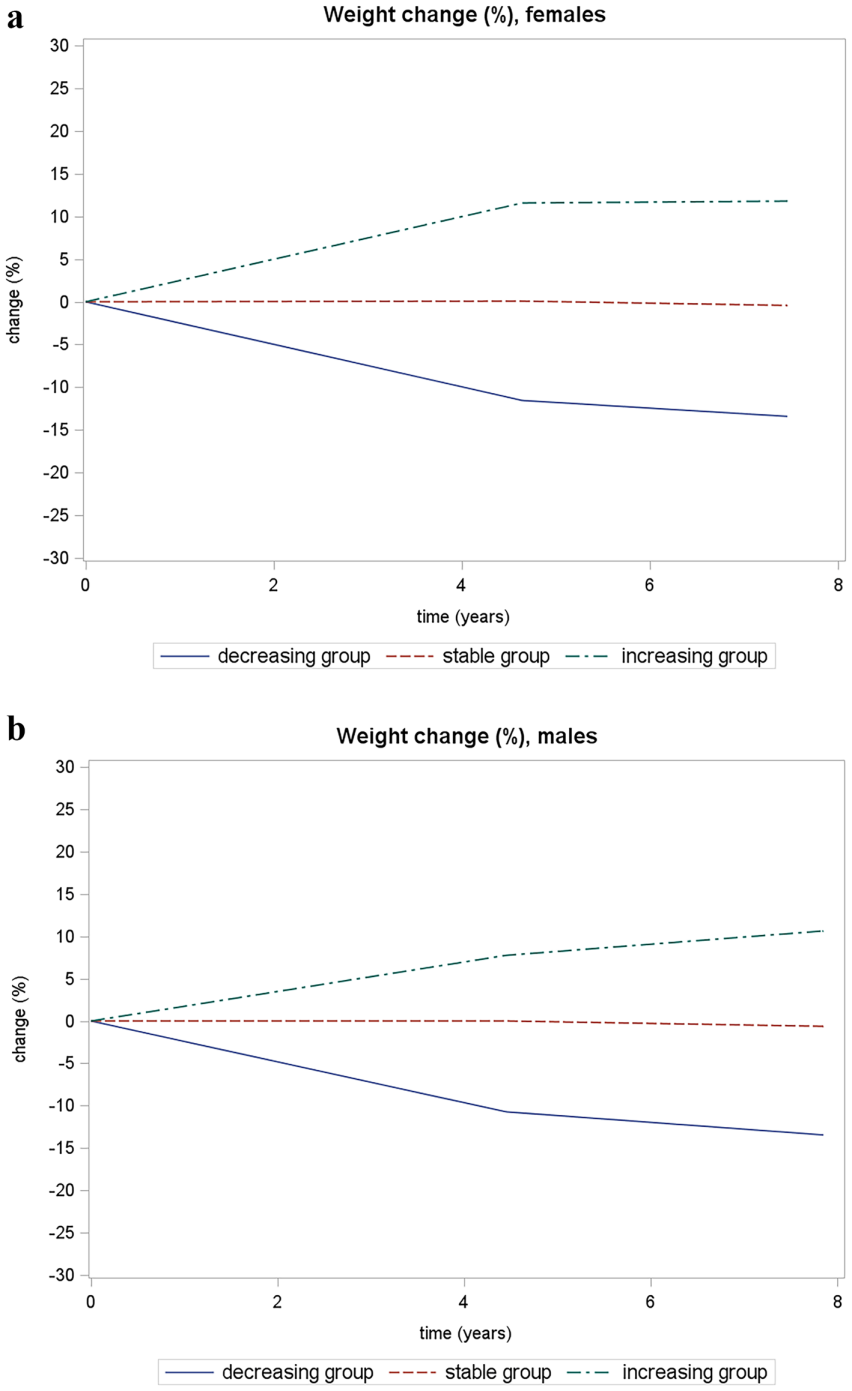
This figure describes weight trajectories identified from baseline to the follow-up, by sex. Value “0” for time (years) axes represent the baseline. Females: BIC-16601.13; probability of “*decreasing group*” membership (average posterior probability, as a measure of entropy) 12.7% (95% CI 11.4–14%), probability of “*stable group*” membership 78.8% (95% CI 77.3–80.4%), probability of “*increasing group*” membership 8.4% (95% CI 7.4–9.5%). Males: BIC-13369.51; probability of “*decreasing group*” membership (average posterior probability, as a measure of entropy) 7.5% (95% CI 7–9.1%), probability of “*stable group*” membership 78.5% (95% CI 77–80.1%), probability of “*increasing group*” membership 13.5% (95% CI 12.2–14.8%). Mean body weight values for: *decreasing group* (males: 76.4 kg at baseline, 67.2 kg at the first follow-up and 66.7 kg at the second; females: 68.6 kg at baseline, 59.6 kg at the first and 58.6 kg at the second follow-up); *stable group* (males: 74.4 kg at baseline, 74.6 kg at the second follow-up; females: 65.9 kg and 66 kg, respectively); *increasing group* (11.9% for males, 7.9% for females) had an increasing weight that, in particular for females, tended to stabilize after the first follow-up (for males: 68.9 kg at the baseline, 75.1 kg at the first follow-up and 78.9 kg at the second; for females, 60.4 kg at the baseline, 68 kg at the first follow-up and 68.6 kg at the second follow-up).

Percent weight change trajectories from baseline to follow-up were evaluated separately for males and females. Three trajectories were identified for both groups (Fig. 1):

- Decreasing weight trajectory (including 131 [7.5%] males, and 257 [12.3%] females), characterized by weight loss during the follow-up period (for males, mean weight 76.4 kg at baseline and 66.7 kg at the second follow-up, mean percentage body weight change from the baseline to the second follow-up − 12.7%, annual mean body weight change − 1.2 kg; for females, 68.6 kg and 58.6 kg, respectively, mean percentage body weight change from the baseline to the second follow-up − 14.6%, annual mean body weight change − 1.3 kg);
- Stable weight trajectory (including 1417 [80.6%] males, and 1671 [79.8%] females, n = 1671), with essentially stable weight (for males, mean weight 74.4 kg at the baseline and 74.6 kg at the second follow-up; for females 65.9 kg and 66 kg, respectively);
- Increasing weight trajectory (including 210 [11.9%] males, and 166 [7.9%] females), with an increase in body weight that tended to stabilize after the first follow-up, especially for females (for males: 68.9 kg at baseline and 78.9 kg at the second follow-up, mean percentage body weight change from the baseline to the second follow-up + 14.5%, annual mean body weight change + 1.3 kg; for females, 60.4 kg and 68.6 kg, respectively mean percentage body weight change from the baseline to the second follow-up + 13.6%, annual mean body weight change + 1 kg).

Table 1 presents the characteristics of the study participants by sex and identified trajectories; in both among males and females, participants with decreasing weight during follow-up were significantly older and had a higher prevalence of diabetes than those with stable or increasing weight. Only among males a higher prevalence of hypertension and lower MMSE scores at baseline were found in the decreasing weight group. These individuals also had the highest BMI, waist and hip circumference at baseline and a lower prevalence of undernutrition according to GLIM criteria than those in the increasing weight trajectory (8.4% vs 22.3% among males; 14.1% vs 30.9% among females) (Supplementary Table 2). In the decreasing weight group, a decline in the mean MMSE score from baseline to follow-up was observed for both males and females (males from 25.1 ± 4.0 at T0, to 24.2 ± 5.3 at the first follow-up, and to 23.1 ± 7.7 at the second follow-up; females from 24.7 ± 4.2, to 22.8 ± 6.1, and to 23.1 ± 6.7, respectively), while groups with stable weight or increasing weight had relatively stable MMSE scores from baseline to follow-up (Table 1).

**Table 1:** Baseline characteristics of study participants according to the weight trajectories *from the baseline to follow-ups.*

	Males					Females				
	All (n = 1758)	Decreasing weight trajectory (n = 131)	Stable weight trajectory (n = 1417)	Increasing weight trajectory (n = 210)	p-value	All (n = 2094)	Decreasing weight trajectory (n = 257)	Stable weight trajectory (n = 1671)	Increasing weight trajectory (n = 166)	p-value
Age at the baseline, mean ± SD	73.5 ± 6.1	75.6 ± 6.3	73.5 ± 6.1	72.7 ± 5.7	< 0.0001	73.8 ± 6.3	75.8 ± 6.1	73.7 ± 6.4	72.5 ± 5.7	< 0.0001
Education, n (%)					0.2334					0.3705
Primary school or less	1285 (73.2)	107 (82.3)	1023 (72.2)	155 (73.8)		1785 (85.4)	220 (85.6)	1426 (85.5)	139 (83.7)	
Middle school	210 (12.0)	11 (8.5)	170 (12.0)	29 (13.8)		163 (7.8)	25 (9.7)	123 (7.4)	15 (9.1)	
High school	145 (8.2)	7 (5.4)	123 (8.7)	15 (7.2)		96 (4.6)	7 (2.7)	83 (5.0)	6 (3.6)	
University or higher	116 (6.6)	5 (3.8)	100 (7.1)	11 (5.2)		46 (2.2)	5 (2.0)	35 (2.1)	6 (3.6)	
Work done for most of the time, n (%)					0.1462					0.0649
Housewife	0 (0.0)	0 (0.0)	0 (0.0)	0 (0.0)		557 (27.3)	64 (25.7)	438 (27.0)	55 (33.5)	
Blue collar	1217 (69.3)	99 (76.2)	968 (68.4)	150 (71.4)		931 (45.7)	118 (47.4)	756 (46.6)	57 (34.8)	
White collar	538 (30.7)	31 (23.8)	447 (31.6)	60 (28.6)		549 (27.0)	67 (26.9)	430 (26.4)	52 (31.7)	
Marital status, n (%)					0.5519					0.5037
Single or never married	80 (4.6)	9 (6.9)	63 (4.4)	8 (3.8)		166 (7.9)	15 (5.8)	137 (8.2)	14 (8.4)	
Married or cohabiting	1428 (81.3)	97 (74.6)	1156 (81.6)	175 (83.7)		885 (42.3)	98 (38.1)	714 (42.7)	73 (44.0)	
Separated or divorced	10 (0.6)	1 (0.8)	8 (0.6)	1 (0.5)		12 (0.6)	2 (0.8)	9 (0.5)	1 (0.6)	
Widowed	238 (13.5)	23 (17.7)	190 (13.4)	25 (12.0)		1031 (49.2(	142 (55.3)	811 (48.6)	78 (47.0)	
SES^§^, n (%)					0.1756					0.6310
1 low	1200 (68.3)	98 (74.8)	954 (67.3)	148 (70.5)		1511 (72.2)	188 (73.1)	1210 (72.5)	113 (68.1)	
2 medium	317 (18.0)	23 (17.6)	256 (18.1)	38 (18.1)		42 (22.6)	59 (23.0)	372 (22.3)	42 (25.3)	
3 high	241 (13.7)	10 (7.6)	207 (14.6)	24 (11.4)		109 (5.2)	10 (3.9)	88 (5.3)	11 (6.6)	
Smoking status, n (%)					0.0207					0.7870
Current smoker	321 (18.3)	18 (13.7)	248 (17.5)	55 (26.2)		122 (5.8)	15 (5.8)	95 (5.7)	12 (7.3)	
Former smoker	1058 (60.3)	85 (64.9)	856 (60.5)	117 (55.7)		189 (9.0)	19 (7.4)	156 (9.3)	14 (8.4)	
Never smoker	377 (21.4)	28 (21.4)	311 (22.0)	38 (18.1)		1782 (85.1)	223 (86.8)	1419 (85.0)	140 (84.3)	
Alcohol consumption, n (%)					0.2877					0.8516
Heavy consumer*	499 (28.4)	43 (32.8)	390 (27.5)	66 (31.6)		169 (8.1)	24 (9.4)	131 (7.8)	14 (8.4)	
Light consumer**	655 (37.3)	38 (29.8)	540 (38.1)	76 (36.3)		430 (20.5)	52 (20.2)	348 (20.8)	30 (18.1)	
No consumer	603 (34.3)	49 (37.4)	487 (34.4)	67 (32.1)		1495 (71.4)	181 (70.4)	1192 (71.4)	122 (73.5)	
Health status variables										
Hypertension, n (%)	1131 (64.3)	94 (71.8)	926 (65.4)	111 (52.9)	0.0004	1493 (71.4)	191 (74.3)	1193 (71.5)	109 (65.7)	0.1534
Diabetes, n (%)	218 (12.4)	26 (19.9)	171 (12.1)	21 (10.0)	0.0192	273 (13.0)	50 (19.5)	205 (12.3)	18 (10.8)	0.0043
Hyperlipidemia, n (%)	517 (30.5)	40 (30.8)	419 (30.8)	58 (28.0)	0.7202	889 (44.5)	96 (37.9)	724 (45.6)	69 (44.0)	0.0762
CVD, n (%)	629 (41.8)	47 (42.3)	508 (41.9)	74 (40.4)	0.9236	472 (25.8)	74 (32.5)	362 (24.8)	36 (25.4)	0.0482
Depressive symptoms, n (%)	364 (21.4)	34 (26.6)	281 (20.5)	49 (23.8)	0.1895	808 (40.7)	110 (44.2)	633 (40.1)	65 (40.6)	0.4792
Walking disability, n (%)	18 (1.0)	1 (0.8)	13 (0.9)	3 (1.4)	0.7712	61 (3.0)	4 (1.6)	50 (3.0)	7 (4.2)	0.2770
MMSE at baseline, mean ± SD	26.3 ± 3.5	25.1 ± 4.0	26.4 ± 3.4	26.0 ± 3.6	< 0.0001	24.9 ± 4.3	24.7 ± 4.2	24.9 ± 4.4	25.3 ± 3.7	0.1423
MMSE at follow-up 1, mean ± SD	25.3 ± 5.3	24.2 ± 5.3	25.3 ± 5.5	25.7 ± 3.7	0.0338	23.3 ± 6.5	22.8 ± 6.1	23.3 ± 6.7	24.3 ± 4.8	0.0966
MMSE at follow-up 2, mean ± SD	26.0 ± 4.7	23.1 ± 7.7	26.3 ± 4.4	25.6 ± 3.8	< 0.0001	24.4 ± 6.0	23.1 ± 6.7	24.5 ± 6.0	25.6 ± 4.2	0.0229
Undernutrition according to GLIM, n (%)	221 (12.6)	11 (8.4)	164 (11.6)	46 (22.3)	< 0.0001	319 (15.3)	36 (14.1)	232 (13.9)	51 (30.9)	< 0.0001
BMI, mean ± SD	26.8 ± 3.7	28.0 ± 4.2	26.9 ± 3.6	25.2 ± 3.5	< 0.0001	28.1 ± 5.0	29.1 ± 5.0	28.2 ± 4.9	25.8 ± 5.0	< 0.0001
BMI, classes, n (%)					< 0.0001					< 0.0001
< 18.5 kg/m^2^	14 (0.8)	0 (0.0)	10 (0.7)	4 (1.9)		25 (1.2)	3 (1.2)	15 (0.9)	7 (4.2)	
18.5–24.9 kg/m^2^	543 (31.1)	31 (23.7)	411 (29.3)	101 (48.1)		537 (25.9)	41 (16.0)	422 (25.6)	74 (44.6)	
25–29.9 kg/m^2^	889 (51.0)	63 (48.1)	738 (52.6)	88 (41.9)		873 (42.1)	108 (42.0)	708 (42.9)	57 (34.3)	
≥ 30 kg/m^2^	299 (17.1)	37 (28.2)	245 (17.4)	17 (8.1)		638 (30.8)	105 (40.8)	505 (30.6)	28 (16.9)	

^§^SES composite measure defined considering education (primary school or middle school: score 1; high school or university or more: score 2) and work done for most of time (blue collar or housewife: score 1; white collar: score 2). Total score 1,2 corresponded to SES = 1 (low); total score 3 to SES = 2 (medium); total score 4 to SES = 3 (high).

*≥ 7 AU/week females; ≥ 14 AU/week males.

**< 7 AU/week females; < 14 AU/week males.

CVD, cardiovascular diseases; MMSE, Mini-Mental State Examination; SD, Standard Deviation; SES, Socioeconomic Status; na, variable not available.

In Cox proportional hazard regression models, taking the stable weight group as the reference, we found no significant association of the decreasing or increasing trajectories from baseline to follow-up with cognitive decline among males or females (Table 3). Results were confirmed when defining cognitive decline based on Minimum Clinically Important Difference for MMSE (Supplementary Table 3) and after performing multiple imputations for missing data (Supplementary Table 4).

**Fig. 2:**
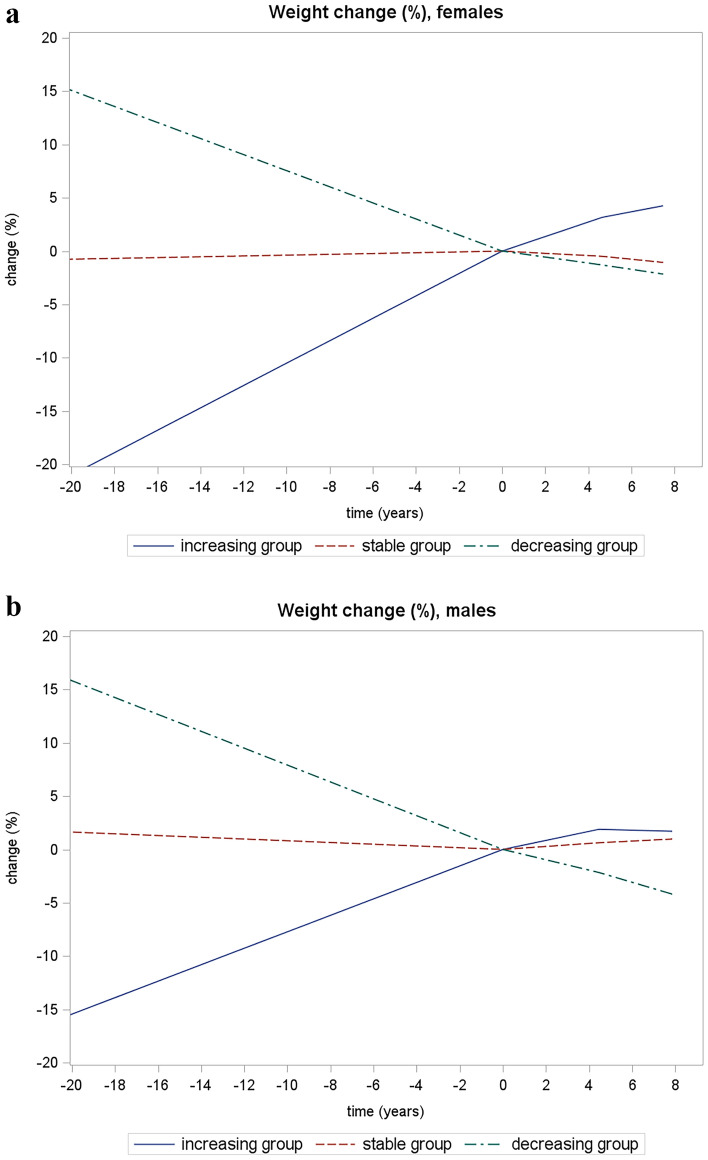
This figure shows weight trajectory identified from age 50 to the second follow-up. Value “0” for time (years) axes represent the baseline. Females: BIC-25120.76; probability of “*decreasing group*” membership (average posterior probability, as a measure of entropy) 35.9% (95% CI 34.3–37.5%), probability of “*stable group*” membership 9.8% (95% CI 8.7–10.8%), probability of “*increasing group*” membership 54.3% (95% CI 52.7–55.9%). Males: BIC-19575.88; probability of “*decreasing group*” membership (average posterior probability, as a measure of entropy) 14.1% (95% CI 12.8–15.3%), probability of “*stable group*” membership 64% (95% CI 62.4–65.7%), probability of “*increasing group*” membership 21.9% (95% CI 20.4–23.4%).

We also evaluated trajectories considering weight at age 50 as the first observation. The following three trajectories were identified for males and females (Fig. 2).

- Increasing weight trajectory (including 182 [17.5%] males, and 631 [30.1%] females), with a more pronounced increase in body weight by baseline (mean weight at 50 years, baseline and the second follow-up: 66.9 kg, 79.4 kg, and 82.6 kg for males; 59.6 kg, 71.2 kg, and 73.2 kg for females).
- Stable weight trajectory (including 1269 [72.2%] males, and 1289 [61.6%] females) with a mean weight at 50 years, baseline and the second follow-up of 71.8 kg, 72.9 kg, and 74 kg for males; and 63.5 kg, 63.3 kg, and 63 kg for females.
- Decreasing weight trajectory (including 307 [10.3%] males, and 174 [8.3%] females), with a more pronounced reduction in body weight more by baseline (mean weight at 50 years, baseline and the second follow-up: 77.6 kg, 66.4 kg, and 65.5 kg for males; 70.6 kg, 60.1 kg, and 56.9 kg for females).

Individuals in the increasing weight trajectory from age 50 were younger than those in the decreasing trajectory (73.4 ± 6.2 and 73.1 ± 6.1 vs. 75.1 ± 6.3 and 75.8 ± 7.0 years for males and females, respectively; Table 2). Males in the increasing weight trajectory were more likely to have low SES, heavy alcohol consumption, hypertension, and diabetes. An opposite trend was observed among females since those in the increasing weight trajectory had lower alcohol consumption and prevalence of diabetes, hyperlipidemia, and CVD compared with the decreasing weight group. In both sexes, subjects with decreasing weight since middle age had the lowest prevalence of obesity, the lowest waist, hips and mid-upper arm circumferences, and the highest prevalence of undernutrition according to the GLIM criteria (42.0% and 40.8%, respectively, for males and females in the decreasing weight trajectory vs 3.0% and 7.1%, respectively, for males and females in the increasing weight trajectory) (Supplementary Table 5). Overall, individuals in the increasing weight trajectory had a mean annual increase in BMI of 0.9% from age 50 to baseline (for males, from 24.1 ± 3.1 kg/m^2^ at age 50 to 29.8 ± 3.7 kg/m^2^ at baseline; for females, from 25.1 ± 3.7 kg/m^2^ at age 50 to 30.7 ± 4.7 kg/m^2^ at baseline). Males in the decreasing weight trajectory had a mean annual decrease in BMI of 0.7% from age 50 to baseline (from 28.2 ± 4.1 kg/m^2^ at age 50 to 23.8 ± 3.2 kg/m^2^ at baseline), whereas females had a mean annual decrease in BMI of 0.9% (from 30.7 ± 5.4 kg/m^2^ at age 50 to 24.8 ± 4.3 kg/m^2^ at baseline).

**Table 2:** Baseline characteristics of study participants according to the weight trajectories from 50 years of age to follow-ups.

	Males									Females						
	All (n = 1758)	Increasing weight trajectory (n = 307)		Stable weight trajectory (n = 1269)		Decreasing weight trajectory (n = 182)			p-value	All (n = 2094)		Increasing weight trajectory (n = 631)	Stable weight trajectory (n = 1289)		Decreasing weight trajectory (n = 174)	p-value
Age at the baseline, mean ± SD	73.5 ± 6.1	73.4 ± 6.2		73.3 ± 6.0		75.1 ± 6.3			0.0012	73.8 ± 6.3		73.1 ± 6.1	73.9 ± 6.3		75.8 ± 7.0	< 0.0001
Education, n (%)									0.1045							0.5484
Primary school or less	1285 (73.2)	239 (77.8)		911 (71.9)		135 (74.2)				1785 (85.4)		546 (86.5)	1087 (84.5)		152 (87.9)	
Middle school	210 (12.0)	40 (13.0)		150 (11.8)		20 (11.0)				163 (7.8)		46 (7.3)	103 (8.0)		14 (8.1)	
High school	145 (8.2)	14 (4.6)		115 (9.1)		16 (8.8)				96 (4.6)		29 (4.6)	62 (4.8)		5 (2.9)	
University or higher	116 (6.6)	14 (4.6)		91 (7.2)		11 (6.0)				46 (2.2)		10 (1.6)	34 (2.6)		2 (1.1)	
Work done for most of the time, n (%)									0.0853							0.3235
Housewife	0 (0.0)	0 (0.0)		0 (0.0)		0 (0.0)				557 (27.3)		155 (25.1)	347 (27.7)		55 (32.9)	
Blue collar	1217 (69.3)	227 (73.9)		872 (68.9)		118 (64.8)				931 (45.7)		296 (47.9)	564 (45.1)		71 (42.5)	
White collar	538 (30.7)	80 (26.1)		394 (31.1)		64 (35.2)				549 (27.0)		167 (27.0)	341 (27.2)		41 (24.6)	
Marital status, n (%)									0.0141							0.2483
Single or never married	80 (4.6)	21 (6.8)		49 (3.9)		10 (5.5)				166 (7.9)		37 (5.9)	114 (8.8)		15 (8.6)	
Married or cohabiting	1428 (81.3)	238 (77.5)		1056 (83.4)		134 (73.6)				885 (42.3)		272 (43.1)	549 (42.6)		64 (36.8)	
Separated or divorced	10 (0.6)	2 (0.7)		6 (0.4)		2 (1.1)				12 (0.6)		3 (0.5)	8 (0.6)		1 (0.6)	
Widowed	238 (13.5)	46 (15.0)		156 (12.3)		36 (19.8)				1031 (49.2)		319 (50.4)	618 (48.0)		94 (54.0)	
SES^§^, n (%)									0.0059							0.5415
1 Low	1200 (68.3)	226 (73.6)		860 (67.7)		114 (62.6)				1511 (72.2)		455 (72.1)	926 (71.8)		130 (75.1)	
2 Medium	317 (18.0)	54 (17.6)		218 (17.2)		45 (24.8)				42 (22.6)		146 (23.1)	289 (22.5)		38 (22.0)	
3 High	241 (13.7)	27 (8.8)		191 (15.1)		23 (12.6)				109 (5.2)		30 (4.8)	74 (5.7)		5 (2.9)	
Smoking status, n (%)									0.8148							0.2955
Current smoker	321 (18.3)	52 (16.9)		232 (18.3)		37 (20.4)				122 (5.8)		36 (5.7)	72 (5.6)		14 (8.1)	
Former smoker	1058 (60.3)	193 (62.9)		759 (59.9)		106 (58.6)				189 (9.0)		57 (9.1)	123 (9.5)		9 (5.1)	
Never smoker	377 (21.4)	62 (20.2)		277 (21.8)		38 (21.0)				1782 (85.1)		537 (85.2)	1094 (84.9)		151 (86.8)	
Alcohol consumption, n (%)									0.0014							0.0070
Heavy consumer*	499 (28.4)	114 (37.1)		347 (27.4)		38 (20.9)				169 (8.1)		33 (5.2)	122 (9.5)		14 (8.1)	
Light consumer**	655 (37.3)	96 (31.3)		483 (38.1)		76 (41.8)				430 (20.5)		124 (19.7)	277 (21.5)		29 (16.7)	
No consumer	603 (34.3)	97 (31.6)		438 (34.5)		68 (37.3)				1495 (71.4)		474 (75.1)	890 (69.0)		131 (75.3)	
Health status variables																
Hypertension, n (%)	1131 (64.3)	237 (77.2)		794 (62.6)		100 (55.0)			< 0.0001	1493 (71.4)		466 (73.9)	899 (69.9)		128 (73.6)	0.1525
Diabetes, n (%)	218 (12.4)	53 (17.3)		139 (11.0)		26 (14.4)			0.0074	273 (13.0)		98 (15.5)	139 (10.8)		36 (20.7)	0.0001
Hyperlipidemia, n (%)	517 (30.5)	103 (34.5)		369 (30.2)		45 (25.3)			0.1037	889 (44.5)		246 (40.0)	566 (46.6)		77 (45.8)	0.0270
CVD, n (%)	629 (41.8)	115 (42.3)		441 (41.2)		73 (44.8)			0.6725	472 (25.8)		120 (21.6)	304 (27.0)		48 (32.2)	0.0105
Depressive symptoms, n (%)	364 (21.4)	64 (21.5)		253 (20.6)		47 (26.6)			0.1977	808 (40.7)		246 (39.7)	486 (40.5)		76 (45.8)	0.3548
Walking disability, n (%)	18 (1.0)	3 (1.0)		12 (1.0)		2 (1.1)			0.9818	61 (3.0)		15 (2.4)	38 (3.0)		8 (4.7)	0.2889
MMSE at baseline, mean ± SD	26.3 ± 3.5	25.8 ± 3.6		26.4 ± 3.5		26.2 ± 3.3			0.7411	24.9 ± 4.3		25.0 ± 4.1	24.9 ± 4.4		24.7 ± 4.1	0.3199
MMSE at follow-up 1, mean ± SD	25.3 ± 5.3	24.7 ± 5.3		25.5 ± 5.2		24.5 ± 5.5			0.0080	23.3 ± 6.5		23.4 ± 6.0	23.5 ± 6.7		21.8 ± 6.9	0.0073
MMSE at follow-up 2, mean ± SD	26.0 ± 4.7	25.5 ± 5.2		26.2 ± 4.4		25.2 ± 5.4			0.2005	24.4 ± 6.0		23.9 ± 6.8	24.7 ± 5.6		24.2 ± 6.4	0.2548
Undernutrition according to GLIM, n (%)	221 (12.6)	9 (3.0)		136 (10.8)		76 (42.0)			< 0.0001	319 (15.3)		45 (7.1)	203 (15.8)		71 (40.8)	< 0.0001

^§^SES composite measure defined considering education (primary school or middle school: score 1; high school or university or more: score 2) and work done for most of time (blue collar or housewife: score 1; white collar: score 2). Total score 1,2 corresponded to SES = 1 (low); total score 3 to SES = 2 (medium); total score 4 to SES = 3 (high).

*≥ 7 AU/week females; ≥ 14 AU/week males.

**< 7 AU/week females; < 14 AU/week males.

CVD, cardiovascular diseases; MMSE, Mini-Mental State Examination; SD, Standard Deviation; SES, Socioeconomic Status.

The decreasing weight trajectory from age 50 was associated with higher likelihood of cognitive decline on the MMSE for both males and females (HR 1.44, 95% CI 1.06–1.94, p = 0.019; HR 1.37, 95% CI 1.12–1.67, p = 0.002, respectively), compared with the stable one. Only in females, the increasing weight trajectory group from age 50 was associated with higher likelihood of cognitive decline during follow-up (HR 1.33, 95% CI 1.01–1.76, p = 0.049) (Table 3). Results were confirmed when defining cognitive decline based on Minimum Clinically Important Difference for MMSE (Supplementary Table 3) but not when considering models with multiple imputations for missing data (Supplementary Table 4), in particular among females. Weight trajectories were also substantially confirmed for both males and females when considering age instead of follow-up years as the time scale (Supplementary Fig. 2). Additional sensitivity analyses evaluated group-based trajectories adjusting for age and sex, and then tested the interaction between sex and defined trajectories; no significant interaction between sex and group was found in models for weight trajectories from baseline to follow-ups (p = 0.723) and from age 50 to follow-ups (p = 0.678) (Supplementary Figs. 3, 4).

**Table 3:** Association between weight trajectories and decline on the MMSE score, by sex.

	Males			Females		
	HR	95% CI	p-value	HR	95% CI	p-value
From baseline to follow-ups						
(a) Unadjusted model						
Stable weight	Ref			Ref		
Increasing weight	0.90	0.66–1.24	0.5240	0.94	0.68–1.31	0.7286
Decreasing weight	1.44	1.03–2.02	0.0327	1.37	1.08–1.74	0.0091
(b) Model adjusted for socio-demographic characteristics						
Stable weight	Ref			Ref		
Increasing weight	0.92	0.68–1.26	0.4400	1.13	0.82–1.57	0.4526
Decreasing weight	1.14	0.81–1.61	0.1153	1.16	0.92–1.48	0.2139
(c) Fully adjusted model						
Stable weight	Ref			Ref		
Increasing weight	0.93	0.67–1.28	0.6450	1.11	0.94–1.52	0.5549
Decreasing weight	1.16	0.82–1.63	0.4093	1.20	0.79–1.55	0.1457
From 50 years of age to follow-ups						
(a) Unadjusted model						
Stable weight	Ref			Ref		
Increasing weight	1.18	0.91–1.52	0.2209	1.28	1.06–1.53	0.0095
Decreasing weight	1.68	1.27–2.22	0.0003	1.58	1.20–2.07	0.0010
(b) Model adjusted for socio-demographic characteristics						
Stable weight	Ref			Ref		
Increasing weight	1.10	0.85–1.42	0.4714	1.34	1.02–1.76	0.0368
Decreasing weight	1.47	1.11–1.95	0.0078	1.30	1.09–1.57	0.0047
(c) Fully adjusted model						
Stable weight	Ref			Ref		
Increasing weight	1.19	0.90–1.56	0.2170	1.33	1.01–1.76	0.0489
Decreasing weight	1.44	1.06–1.94	0.0190	1.37	1.12–1.67	0.0023

CI, Confidence Interval; HR, Hazard Ratio.

Models (b) were adjusted for age, education, work done, and marital status.

Models (c) were adjusted for age, education, work done, marital status, smoking status, alcohol consumption, BMI, diabetes, hyperlipidemia, cardiovascular diseases, depression, limited mobility and baseline MMSE score.

## Discussion

Our study examined the possible associations between body weight trajectories over the years and cognitive decline. Three main findings can be summarized from our data: (1) body weight trajectories are associated with cognitive decline in advancing age; (2) people experiencing a decrease in body weight from middle to old age have a steeper cognitive decline; (3) only among females, an increase in body weight from middle age was adversely associated with cognitive health. These results underscore the importance of considering body weight trajectories not only when assessing physical health but also for long-term cognitive health. Moreover, our findings call for further research to develop sex and gender-specific tailored interventions and to deepen the understanding of the biological mechanisms linking variations in body weight to cognitive decline.

In older adults, variations in body weight reflect changes in organs and tissues, including the simultaneous redistribution of fat and lean mass that naturally accompanies aging^41–43^. The existence of these changes prompted researchers to evaluate whether low or excess weight could impact health status differently in advanced than at younger age. As summarized in a meta-analysis involving about 200,000 older individuals, BMI values either below 23 kg/m^2^ or above 33 kg/m^2^ were associated with an increased risk of mortality^41,44^, fostering the concept of “obesity paradox”.

Focusing on changes in body weight in older age, we did not find any significant association between decreasing or increasing weight trajectories and cognitive decline regardless of biological sex, although individuals with weight loss had a poorer health status and were more likely to be undernourished. Conversely, when analyzing weight trajectories since age 50, a noteworthy association between weight loss and cognitive decline was observed in both sexes. Moreover, only among females, those who increased weight since age 50 were more likely to decline in cognitive function than those who were stable. In our study, we found that in both sexes, individuals who experienced weight loss since middle age tended to have a lower prevalence of obesity and smaller waist and hip circumferences, implying a reduction in body fat. These individuals also had a high prevalence of undernutrition and smaller mid-upper arm circumference, suggesting that their weight loss was due to a reduction not only in fat but also in muscle mass. Interestingly, we also observed that the group of females who gained weight were more likely to have lower alcohol consumption and prevalence of diabetes, hyperlipidemia, and cardiovascular diseases compared to those experiencing weight loss. Despite these apparently healthier characteristics, our results may reflect what is already known, namely that females are more likely to increase their proportion of body fat from adulthood^45^, while the changes in body composition observed in males are predominantly determined by lean mass changes^46,47^. The aetiology of age-related loss of muscle mass and strength—commonly referred to as sarcopenia—is multifactorial and complex, with emerging evidence suggesting that it may be sex-specific^48^. Indeed, differences in the prevalence and functional manifestations of sarcopenia have been reported between males and females, with males often experiencing higher rates of muscle mass loss during aging^48^. Sarcopenia has been shown to impact cognition through mechanisms such as reduced mobility, which diminishes cerebral blood flow and the production of neurotrophic factors (e.g., brain-derived neurotrophic factor) that are essential for neuronal health, as well as through disrupted muscle–brain crosstalk, where weakened skeletal muscles produce fewer myokines that regulate brain metabolism and synaptic plasticity^49^. Concurrently, the accumulation of adipose tissue adversely affects cognitive health, primarily by elevating leptin levels and provoking inflammation, which compromises brain metabolism and accelerates neuronal degradation^50^. Excess adiposity elevates systemic leptin concentrations, yet resistance to leptin signaling further undermines both metabolic and cognitive health. For instance, hypothalamic leptin resistance disrupts energy homeostasis and promotes obesity and peripheral insulin resistance, and hippocampal leptin resistance may impair synaptic plasticity and memory formation while fostering amyloid-beta accumulation^51^. Notably, age-related adiposity increases in females could lead to greater leptin exposure, thereby exacerbating resistance and amyloid-beta pathology compared to males^51^. Finally, adipose tissue-derived inflammation is also pivotal in driving cognitive decline through multiple interconnected pathways. Excessive adiposity, particularly visceral fat, instigates chronic low-grade systemic inflammation by releasing pro-inflammatory cytokines such as TNF-α, IL-6, and leptin^52^. These inflammatory mediators can cross the blood–brain barrier via the vagus nerve or as a result of compromised barrier integrity, activating microglia in critical brain regions such as the hippocampus and hypothalamus. Once in the central nervous system, TNF-α and IL-6 disrupt insulin signaling pathways that are essential for synaptic plasticity and memory formation^52^. The resulting microglial activation perpetuates a cycle of neuroinflammation, oxidative stress, diminished neurotrophic factor production, and neuronal degradation^52^. Sex differences further emerge in these processes: males with higher levels of visceral adipose tissue exhibit stronger associations between inflammation and cognitive deficits—as evidenced by markers like CRP and IL-6—whereas females’ greater adiposity, despite increasing leptin exposure, may be partially mitigated by estrogens’ anti-inflammatory effects^53^. Ultimately, this inflammatory cascade contributes to white matter damage, reduced cortical volume, and impaired executive function, thereby creating a self-reinforcing cycle that accelerates neurodegeneration. So far, several studies have attempted to investigate the association between weight or BMI changes and cognitive decline. However, only a few have examined sex differences, and results are often contradictory^26,54–58^. Although our study focused on cognitive decline rather than dementia, our findings align with previous research indicating that cognitive decline is more frequent in individuals who experience progressive weight loss in older age^57,58^. In particular, the Whitehall II study showed that BMI trajectories from age 50 differed from those at subsequent ages ^57^ and that from 16 to 28 years before diagnosis, dementia cases had a higher BMI than controls and were more likely to be obese at age 50. Still, their BMI decreased below that of controls about 8 years before the diagnosis of the disease^57^. This phenomenon does not seem to be entirely explained by undernutrition, which appears to affect more physical function than cognitive status^54^. Similarly, several authors reported that weight loss begins years before the onset of clinical dementia, with a progressive occurrence of cognitive deficits, impairments in daily activities, loss of olfactory and gustatory functions, decreased physical activity and food intake, which result in weight loss or less weight gain^59–61^. Additionally, psychosocial changes during aging may induce behavioral variations, triggering a vicious cycle^58^. For example, the loss of social roles and relationships (such as retirement or widowhood), decreased autonomy, social isolation, and loneliness can lead to increased irritability, apathy, difficulty adapting to changes, reduced motivation and physical activity, and the adoption of unhealthy lifestyles. The loss of social roles can decrease appetite and interest in food, making meals less enjoyable and social, resulting in reduced nutritional intake. Decreased autonomy makes it difficult to shop, cook, and eat properly, leading to monotonous and inadequate diets. Additionally, changes in mood and mental health can negatively affect appetite and eating habits, contributing to malnutrition^58,62,63^.

Of note, when interpreting the sex differences observed in our data, the different number of participants and the extent to which body weight changed should be taken into account. In fact, weight-increasing trajectories from adulthood included a nearly double proportion of females compared to males, and the former also showed a greater extent of weight increase from age 50. Moreover, sex-specific trajectories in cognitive decline should be interpreted through a multidimensional lens that includes biological, hormonal, and sociocultural factors. The decline in estrogen levels during and after menopause, for example, may accelerate the accumulation of visceral adipose tissue in females and increase their susceptibility to leptin resistance and systemic inflammation^64^, both of which—as previously mentioned—are implicated in neurodegeneration. Estrogens have well-documented neuroprotective properties—mediated through anti-inflammatory, antioxidant, and synaptic plasticity-enhancing effects—and their loss may therefore contribute to the greater vulnerability of older females to adiposity-related cognitive decline^65^. Additionally, differences in fat distribution—peripheral in premenopausal females and central in males—tend to converge with aging, leading to increased visceral adiposity in females later in life^45^. This shift may partly account for the adverse association observed between weight gain and cognitive health in the females included in our study. Beyond biological differences, social and behavioral factors may also play a critical role. For instance, older females often face a higher burden of caregiving responsibilities^66^, greater risk of social isolation^67^, and lower socioeconomic status^68^, all of which may influence diet quality, physical activity, stress levels, access to healthcare, and, ultimately, nutritional and cognitive status. These considerations underscore the need for future studies to move beyond binary comparisons and more thoroughly disentangle the complex interplay of biological and social mechanisms that underlie sex disparities in cognitive decline.

This study has several limitations that need to be acknowledged. First, we recognize that body weight alone may be a crude anthropometric measure, especially in older people. Nevertheless, it is an easily accessible and readily measurable parameter, allowing for more accurate intraindividual monitoring. Concerning the retrospectively assessed body weight at age 50, a further limitation can be linked to recall bias. Previous studies evaluating the reliability of short and long-term weight recall in older adults found a strong correlation between reported and measured weight for 20- or 30-year recalls^69,70^, and showed that the underestimation of recalled weight did not exceed 1.89 kg^71^. However, accuracy gradually decreased with aging, in the case of cognitive deficits, and based on current weight^69,71^. In particular, overweight/obese individuals tended to underestimate past weight, while having lower BMI, experiencing larger weight gains, and presenting with poorer cognitive function were associated with an overestimation of past weight^69,71,72^. In our study, these tendencies might have led individuals in the increasing and decreasing trajectories to, respectively, underestimate or overestimate their weight at age 50, thus exacerbating the differences between their weight at midlife and in older age. However, since the differences between baseline and midlife weight for these groups exceed 10 kg, we argue that any possible recall bias would not have changed the participants’ assignment to the increasing or decreasing trajectories. Second, the retrospective study design and data harmonization could not rule out a certain heterogeneity because the included studies differed in their original main objectives, recruitment procedures, and data collection. A significant limitation in the outcome assessment is the use exclusively of the MMSE, one of the most widely employed tools for evaluating global cognitive function. Although the MMSE provides a useful overview of general cognitive function and allows for monitoring the progression of cognitive decline, it has some well-known limitations. For instance, it may not always detect milder forms of cognitive decline or deficits in some specific domains (e.g. executive function). Therefore, it would be interesting to verify our findings in other samples with more comprehensive neuropsychological tests. Finally, some of the trajectories were limited by small sample sizes. The exclusion of approximately half of the participants due to missing data may have introduced selection bias, thereby limiting the generalizability of the findings. Specifically, because the excluded individuals differed in key characteristics—such as health status, comorbidities, or socio-demographic factors—compared to those included, the observed associations may not fully reflect the broader dynamics of the target population. Moreover, the reduced sample size could have compromised the statistical power of the study, hindering the ability to detect meaningful effects and diminishing the reliability of the results. Despite these limitations, the study has several strengths that enhance its value. The harmonization and pooling of data from two Italian observational studies yielded a large, population-based sample, providing a unique resource for epidemiological research on nutritional status and neurocognitive outcomes with greater statistical power. The prospective design of the included studies minimizes bias due to reverse causation, as body weight was assessed before the development of outcomes. Efforts to standardize the harmonization of exposures, outcomes, and confounders reduce potential sources of heterogeneity and enhance comparability.

## Conclusions

Changes in body weight from middle to older age are strongly linked to cognitive decline in advanced age, underscoring the need for monitoring weight trajectories as a potential early indicator of cognitive health. Our findings suggest that both weight loss and, in certain cases, weight gain—particularly among females—may be associated with an accelerated cognitive decline. However, it is essential to recognize that body weight is shaped by a complex array of factors, including metabolic alterations, chronic health conditions, lifestyle behaviors, and social determinants. Given this complexity, a holistic and multifaceted approach is necessary when evaluating the relationship between body weight and cognitive decline. Future research should strive to incorporate these diverse factors, offering a more comprehensive understanding of how fluctuations in body weight impact long-term cognitive outcomes and informing more targeted interventions for aging populations.

## Supplementary Information

Supplementary Information.

## Data Availability

Data will be available upon reasonable request to Marianna Noale (marianna.noale@in.cnr.it).
