# Supplementary material for: Body weight trajectories from midlife are associated with cognitive decline in advanced age
Source: Sci Rep. 2025 Jul 6;15:24128. doi: 10.1038/s41598-025-08725-5 (PMC12230133; doi:10.1038/s41598-025-08725-5)
Supplement: Supplementary file 1 — Supplementary Information. [file 41598_2025_8725_MOESM1_ESM.docx]

**Supplementary Table 1.** Characteristics of study participants included or not included in the analyses

|  | **Included**  **in the analyses**  **(n=3852)** | **Not included**  **in the analyses**  **(n=3615)** | **p-value** |
| --- | --- | --- | --- |
| Age at the baseline, mean±SD | 73.7±6.2 | 76.8±6.5 | <0.0001 |
| Sex, female, n (%) | 2094 (54.4) | 1846 (51.1) | 0.0044 |
| Education, n (%)  Primary school or less  Middle school  High school  University or higher | 3070 (79.8)  373 (9.7)  241 (6.3)  162 (4.2) | 2694 (76.2)  398 (11.3)  301 (8.5)  144 (4.1) | 0.0002 |
| Work done for most of the time, n (%)  Housewife  Blue collar  White collar | 557 (14.7)  2148 (56.6)  1087 (28.7) | 669 (19.6)  1785 (52.3)  957 (28.1) | <0.0001 |
| Marital status, n (%)  Single or never married  Married or cohabiting  Separated or divorced  Widowed | 246 (6.4)  2313 (60.1)  22 (0.6)  1269 (33.0) | 295 (8.2)  1876 (52.3)  42 (1.2)  1373 (38.3) | <0.0001 |
| SES^§^, n (%)  1 low  2 medium  3 high | 2711 (70.4)  790 (20.5)  350 (9.1) | 2537 (70.9)  684 (19.1)  359 (10.0) | 0.1630 |
| *Lifestyle* |  |  |  |
| Smoking status, n (%)  Current smoker  Former smoker  Never smoker | 443 (11.5)  1247 (32.4)  2159 (56.1) | 434 (12.2)  1156 (32.4)  1975 (55.4) | 0.6536 |
| Alcohol consumption, n (%)  Heavy consumer*  Light consumer**  No consumer | 668 (17.4)  1085 (28.2)  2098 (54.4) | 644 (18.1)  1074 (30.1)  1845 (51.8) | 0.0624 |
| *Health status variables* |  |  |  |
| Hypertension, n (%) | 2624 (68.2) | 2252 (63.3) | <0.0001 |
| Diabetes, n (%) | 491 (12.8) | 574 (16.1) | <0.0001 |
| Hyperlipidemia, n (%) | 1406 (38.0) | 918 (27.9) | <0.0001 |
| CVD, n (%) | 1101 (33.0) | 1407 (44.5) | <0.0001 |
| Depressive symptoms, n (%) | 1172 (31.8) | 1016 (46.2) | <0.0001 |
| Walking ability, n (%) | 3706 (97.9) | 1997 (89.0) | <0.0001 |

^§^: SES composite measure defined considering education (primary school or middle school: score 1; high school or university or more: score 2) and work done for most of time (blue collar or housewife: score 1; white collar: score 2). Total score 1,2 corresponded to SES=1 (low); total score 3 to SES=2 (medium); total score 4 to SES=3 (high)

*: ≥7 AU/week females; ≥14 AU/week males

**: <7 AU/week females; <14 AU/week males

*Abbreviations:* CVD, cardiovascular diseases; MMSE, Mini-Mental State Examination; SD, Standard Deviation; SES, Socioeconomic Status; na, variable not available

**Supplementary Table 2.** Variables related to GLIM phenotypic and etiologic criteria according to the weight trajectories from baseline to follow-up

|  | **Men** | | | | | **Females** | | | | |
| --- | --- | --- | --- | --- | --- | --- | --- | --- | --- | --- |
|  | **All**  **(n=1758)** | **Decreasing weight trajectory**  **(n=131)** | **Stable weight trajectory**  **(n=1417)** | **Increasing weight trajectory**  **(n=210)** | **p-value** | **All**  **(n=2094)** | **Decreasing weight trajectory**  **(n=257)** | **Stable weight trajectory**  **(n=1671)** | **Increasing weight trajectory**  **(n=166)** | **p-value** |
| BMI, kg/m^2^, mean±SD | 26.8±3.7 | 28.0±4.2 | 26.9±3.6 | 25.2±3.5 | <0.0001 | 28.1±5.0 | 29.1±5.0 | 28.2±4.9 | 25.8±5.0 | <0.0001 |
| BMI, n (%)  <18.5 kg/m^2^  18.5-24.9 kg/m^2^  25-29.9 kg/m^2^  ≥30 kg/m^2^ | 14 (0.8)  543 (31.1)  889 (51.0)  299 (17.1) | 0 (0.0)  31 (23.7)  63 (48.1)  37 (28.2) | 10 (0.7)  411 (29.3)  738 (52.6)  245 (17.4) | 4 (1.9)  101 (48.1)  88 (41.9)  17 (8.1) | <0.0001 | 25 (1.2)  537 (25.9)  873 (42.1)  638 (30.8) | 3 (1.1)  41 (16.0)  108 (42.0)  105 (40.9) | 15 (0.9)  422 (25.6)  708 (42.9)  505 (30.6) | 7 (4.2)  74 (44.6)  57 (34.3)  28 (16.9) | <0.0001 |
| Waist circumference, cm, mean±SD | 97.5±10.2 | 100±11.3 | 97.8±10.0 | 94.0±10.1 | <0.0001 | 96±12.6 | 98.5±12.6 | 96.1±12.6 | 91.3±11.7 | <0.0001 |
| Mid-upper arm circumference, cm, mean±SD | 30.2±4.6 | 30.3±3.5 | 30.3±4.8 | 29.8±3.0 | 0.6155 | 30.4±4.8 | 30.5±3.5 | 30.5±5.0 | 29.7±3.9 | 0.2654 |
| Hips circumference, cm, mean±SD | 99.8±8.3 | 101.8±9.6 | 99.9±8.2 | 97.7±7.8 | <0.0001 | 102.7±19.1 | 104.4±12.1 | 102.9±20.6 | 98.8±10.8 | 0.0108 |
| Weight baseline, kg, mean±SD | 73.9±12.0 | 76.4±14.9 | 74.4±11.6 | 68.9±11.1 | <0.0001 | 65.8±12.8 | 68.6±15.7 | 65.9±12.2 | 60.4±11.8 | <0.0001 |
| Weight follow-up 1, kg, mean±SD | 73.9±12.0 | 67.2±12.4 | 74.3±11.7 | 75.1±12.1 | <0.0001 | 65.3±12.5 | 59.6±12.0 | 65.9±12.3 | 67.9±13.1 | <0.0001 |
| Weight follow-up 2, kg, mean±SD | 74.6±12.7 | 66.7±13.0 | 74.6±11.9 | 78.9±14.9 | <0.0001 | 65.4±12.4 | 58.6±11.4 | 66.0±12.3 | 68.6±12.3 | <0.0001 |
| Weight at age 50, kg, mean±SD | 71.6±10.6 | 70.5±11.3 | 72.0±10.6 | 69.8±9.5 | 0.0229 | 62.8±10.8 | 64.1±11.5 | 62.8±10.8 | 61.2±10.1 | 0.0504 |
| Weight loss in the last year >5kg, n (%) | 127 (7.3) | 7 (5.4) | 92 (6.6) | 28 (13.5) | 0.0011 | 201 (9.8) | 27 (10.8) | 141 (8.6) | 33 (20.6) | <0.0001 |
|  |  |  |  |  |  |  |  |  |  |  |
| *Variables related to GLIM etiologic criteria* |  |  |  |  |  |  |  |  |  |  |
| Self-reported bowel/stomach diseases, n (%) | 678 (38.7) | 54 (41.2) | 536 (38.0) | 88 (41.9) | 0.4579 | 663 (31.8) | 75 (29.2) | 518 (31.1) | 70 (42.2) | 0.0091 |
| Self-reported liver or gallbladder diseases, n (%) | 355 (20.6) | 18 (14.0) | 292 (21.0) | 45 (21.8) | 0.1493 | 631 (30.3) | 77 (30.1) | 497 (29.9) | 57 (34.6) | 0.4582 |
| Self-reported chronic bronchitis, n (%) | 396 (22.9) | 41 (31.3) | 305 (21.9) | 50 (24.3) | 0.0427 | 222 (10.8) | 34 (13.5) | 168 (10.1) | 20 (12.3) | 0.2195 |
| Self-reported cancer, n (%) | 77 (4.4) | 8 (6.2) | 59 (4.2) | 10 (4.8) | 0.5538 | 130 (6.2) | 15 (5.9) | 105 (6.3) | 10 (6.1) | 0.9665 |
| Self-reported bone diseases, n (%) | 850 (48.5) | 66 (50.4) | 673 (47.7) | 111 (53.1) | 0.3124 | 1558 (74.6) | 185 (72.3) | 1246 (74.8) | 127 (76.5) | 0.5809 |
| Congestive heart failure, n (%) | 88 (5.0) | 5 (3.8) | 74 (5.2) | 9 (4.3) | 0.6845 | 132 (6.3) | 16 (6.3) | 111 (6.6) | 5 (3.0) | 0.1854 |
| Albumin, %, mean±SD | 57.8±22.0 | 57.2±10.7 | 57.3±12.3 | 62.1±54.2 | 0.4533 | 57.7±9.2 | 56.6±10.8 | 57.8±8.6 | 58.5±8.5 | 0.0827 |
| Fibrinogen, mg/dl, mean±SD | 331.1±119.7 | 330.7±91.7 | 333.2±126 | 317.4±88.3 | 0.2156 | 349.2±78.6 | 355.1±79.4 | 347.9±78.7 | 353.4±76.3 | 0.3262 |
| WBC, x1000/mm^3^, mean±SD | 6.1±2.2 | 6.0±1.4 | 6.1±1.7 | 6.3±4.5 | 0.5362 | 5.9±2.2 | 5.9±1.4 | 5.9±2.3 | 57±1.6 | 0.5461 |

*Abbreviations:* SD, Standard Deviation

**Supplementary Table 3.** Association between weight trajectories and decline on the MMSE score defined considering Minimum Clinical Important Difference, by sex

|  | **Males** | | | **Females** | | |
| --- | --- | --- | --- | --- | --- | --- |
|  | **HR** | **95% CI** | **p-value** | **HR** | **95% CI** | **p-value** |
| *From baseline to follow-ups* |  |  |  |  |  |  |
| Stable weight | ref |  |  | ref |  |  |
| Increasing weight | 0.93 | 0.67-1.29 | 0.6617 | 1.11 | 0.79-1.56 | 0.5442 |
| Decreasing weight | 1.16 | 0.83-1.64 | 0.3837 | 1.20 | 0.94-1.53 | 0.1399 |
|  |  |  |  |  |  |  |
| *From 50 years of age to follow-ups* |  |  |  |  |  |  |
| Stable weight | ref |  |  | ref |  |  |
| Increasing weight | 1.17 | 0.89-1.54 | 0.2551 | 1.33 | 1.01-1.77 | 0.0461 |
| Decreasing weight | 1.44 | 1.07-1.95 | 0.0170 | 1.37 | 1.12-1.68 | 0.0021 |

*Abbreviations:* CI, Confidence Interval; HR, Hazard Ratio

Models were adjusted for age, education, work done, marital status, smoking status, alcohol consumption, BMI, diabetes, hyperlipidemia, cardiovascular diseases, depression, limited mobility and MMSE baseline score.

**Supplementary Table 4.** Association between weight trajectories and decline on the MMSE score, by sex (models with multiple imputations for missing data)

|  | **Males** | | | **Women** | | |
| --- | --- | --- | --- | --- | --- | --- |
|  | **HR** | **95% CI** | **p-value** | **HR** | **95% CI** | **p-value** |
| *From baseline to follow-ups* |  |  |  |  |  |  |
| Stable weight | ref |  |  | ref |  |  |
| Increasing weight | 0.96 | 0.69-1.32 | 0.7844 | 1.07 | 0.86-1.33 | 0.5409 |
| Decreasing weight | 1.14 | 0.84-1.53 | 0.3963 | 1.13 | 0.85-1.51 | 0.4054 |
|  |  |  |  |  |  |  |
| *From 50 years of age to follow-ups* |  |  |  |  |  |  |
| Stable weight | ref |  |  | ref |  |  |
| Increasing weight | 1.23 | 0.82-1.85 | 0.3132 | 1.11 | 0.78-1.56 | 0.5657 |
| Decreasing weight | 0.98 | 0.79-1.22 | 0.8823 | 1.02 | 0.86-1.21 | 0.8507 |

*Abbreviations:* CI, Confidence Interval; HR, Hazard Ratio

Models were adjusted for age, education, work done, marital status, smoking status, alcohol consumption, BMI, diabetes, hyperlipidemia, cardiovascular diseases, depression, limited mobility and MMSE baseline score.

**Supplementary Table 5.** Variables related to GLIM phenotypic and etiologic criteria according to the weight trajectories from age 50 to follow-up

|  | **Males** | | | | | **Females** | | | | |
| --- | --- | --- | --- | --- | --- | --- | --- | --- | --- | --- |
|  | **All**  **(n=1758)** | **Increasing weight trajectory**  **(n=307)** | **Stable weight trajectory**  **(n=1269)** | **Decreasing weight trajectory**  **(n=182)** | **p-value** | **All**  **(n=2094)** | **Increasing weight trajectory**  **(n=631)** | **Stable weight trajectory**  **(n=1289)** | **Decreasing weight trajectory**  **(n=174)** | **p-value** |
| BMI, kg/m^2^, mean±SD | 26.8±3.7 | 29.8±3.7 | 26.4±3.3 | 23.8±3.2 | <0.0001 | 28.1±5.0 | 30.7±4.7 | 27.2±4.6 | 24.8±4.3 | <0.0001 |
| BMI, n (%)  <18.5 kg/m^2^  18.5-24.9 kg/m^2^  25-29.9 kg/m^2^  ≥30 kg/m^2^ | 14 (0.8)  543 (31.1)  889 (51.0)  299 (17.1) | 0 (0.0)  25 (8.2)  155 (50.6)  126 (41.2) | 5 (0.4)  413 (32.9)  673 (53.5)  166 (13.2) | 9 (5.0)  105 (57.7)  61 (33.5)  7 (3.8) | <0.0001 | 25 (1.2)  537 (25.9)  873 (42.1)  638 (30.8) | 0 (0.0)  60 (9.5)  244 (38.7)  327 (51.8) | 16 (1.3)  391 (30.8)  570 (44.9)  293 (23.1) | 9 (5.2)  86 (50.0)  59 (34.3)  18 (10.5) | <0.0001 |
| Waist circumference, cm, mean±SD | 97.5±10.2 | 104.9±9.8 | 96.7±9.4 | 90.9±9.5 | <0.0001 | 96±12.6 | 101.5±11.4 | 94.2±12.4 | 89.0±11.6 | <0.0001 |
| Mid-upper arm circumference, cm, mean±SD | 30.2±4.6 | 31.9±2.8 | 30.1±5.0 | 27.8±2.8 | <0.0001 | 30.4±4.8 | 32±3.3 | 29.6±3.4 | 28.8±11.8 | <0.0001 |
| Hips circumference, cm, mean±SD | 99.8±8.3 | 104.8±9.0 | 99.2±7.7 | 95.0±7.3 | <0.0001 | 102.7±19.1 | 107.1±10.9 | 101.1±22.6 | 96.3±9.0 | <0.0001 |
| Weight baseline, kg, mean±SD | 73.9±12.0 | 79.4±14.4 | 72.9±11.9 | 66.4±10.2 | <0.0001 | 65.8±12.8 | 71.2±12.4 | 63.3±11.5 | 60.1±10.3 | <0.0001 |
| Weight follow-up 1, kg, mean±SD | 73.9±12.0 | 79.4±14.4 | 73.4±11.2 | 66.5±10.5 | <0.0001 | 65.3±12.5 | 71.9±12.4 | 63.0±11.4 | 58.7±10.7 |  |
| Weight follow-up 2, kg, mean±SD | 74.6±12.7 | 82.6±13.0 | 74.0±11.9 | 65.5±10.2 | <0.0001 | 65.4±12.4 | 73.2±12.4 | 62.8±11.3 | 56.9±11.5 |  |
| Weight at age 50, kg, mean±SD | 71.6±10.6 | 66.9±9.5 | 71.8±9.7 | 77.6±12.6 | <0.0001 | 62.8±10.8 | 59.6±8.9 | 63.5±10.8 | 70.6±13.0 | <0.0001 |
| Weight loss in the last year >5kg, n (%) | 127 (7.3) | 14 (4.6) | 78 (6.2) | 35 (19.3) | <0.0001 | 201 (9.8) | 43 (6.9) | 115 (9.1) | 43 (25.3) | <0.0001 |
|  |  |  |  |  |  |  |  |  |  |  |
| *Variables related to GLIM etiologic criteria* |  |  |  |  |  |  |  |  |  |  |
| Self-reported bowel/stomach diseases, n (%) | 678 (38.7) | 110 (35.8) | 483 (38.2) | 85 (47.5) | 0.0294 | 663 (31.8) | 162 (25.8) | 437 (34.0) | 64 (36.8) | 0.0004 |
| Self-reported liver or gallbladder diseases, n (%) | 355 (20.6) | 63 (21.0) | 245 (19.6) | 47 (26.3) | 0.1193 | 631 (30.3) | 214 (34.0) | 357 (27.8) | 60 (34.9) | 0.0081 |
| Self-reported chronic bronchitis, n (%) | 396 (22.9) | 84 (27.8) | 274 (21.9) | 38 (21.2) | 0.0772 | 222 (10.8) | 68 (10.9) | 132 (10.4) | 22 (12.6) | 0.6537 |
| Self-reported cancer, n (%) | 77 (4.4) | 15 (4.9) | 48 (3.8) | 14 (7.7) | 0.0479 | 130 (6.2) | 43 (6.9) | 74 (5.8) | 13 (7.6) | 0.5003 |
| Self-reported bone diseases, n (%) | 850 (48.5) | 138 (45.1) | 618 (48.9) | 94 (51.9) | 0.3090 | 1558 (74.6) | 466 (74.0) | 947 (73.8) | 145 (83.3) | 0.0221 |
| Congestive heart failure, n (%) | 88 (5.0) | 23 (7.5) | 52 (4.1) | 13 (7.1) | 0.0189 | 132 (6.3) | 34 (5.4) | 77 (6.0) | 21 (12.1) | 0.0043 |
| Albumin, %, mean±SD | 57.8±22.0 | 57.7±10.4 | 58.1±24.9 | 56.5±13.3 | 0.6587 | 57.7±9.2 | 57.9±8.3 | 57.5±9.9 | 58.3±7.0 | 0.4723 |
| Fibrinogen, mg/dl, mean±SD | 331.1±119.7 | 331.7±84.4 | 331.4±131 | 327.8±82 | 0.9288 | 349.2±78.6 | 350.5±78.4 | 346.9±76.4 | 361.1±93.4 | 0.1519 |
| WBC, x1000/mm^3^, mean±SD | 6.1±2.2 | 6.3±2.2 | 6.1±2.3 | 6.1±1.7 | 0.2899 | 5.9±2.2 | 5.9±1.5 | 5.8±2.5 | 5.9±1.6 | 0.7924 |

*Abbreviations:* SD, Standard Deviation

**Supplementary Figure 1.** Flow-chart of the sample selection and trajectory classification


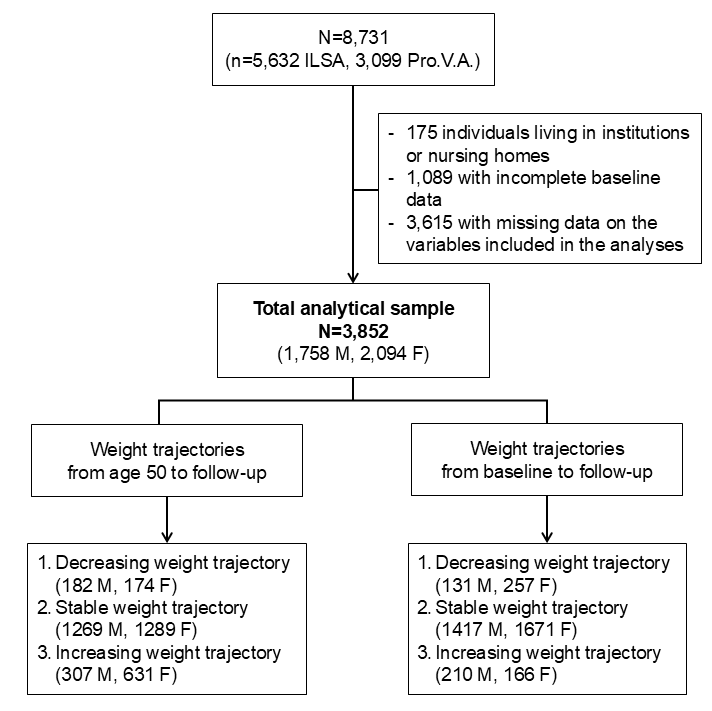


*Abbreviations*: F, females; ILSA, Italian Longitudinal Study of Ageing; M, males; Pro.V.A., Progetto Veneto Anziani.

**Supplementary Figure 2.** Weight trajectory identified from age 50 to the follow-ups, considering chronological age as the time scale


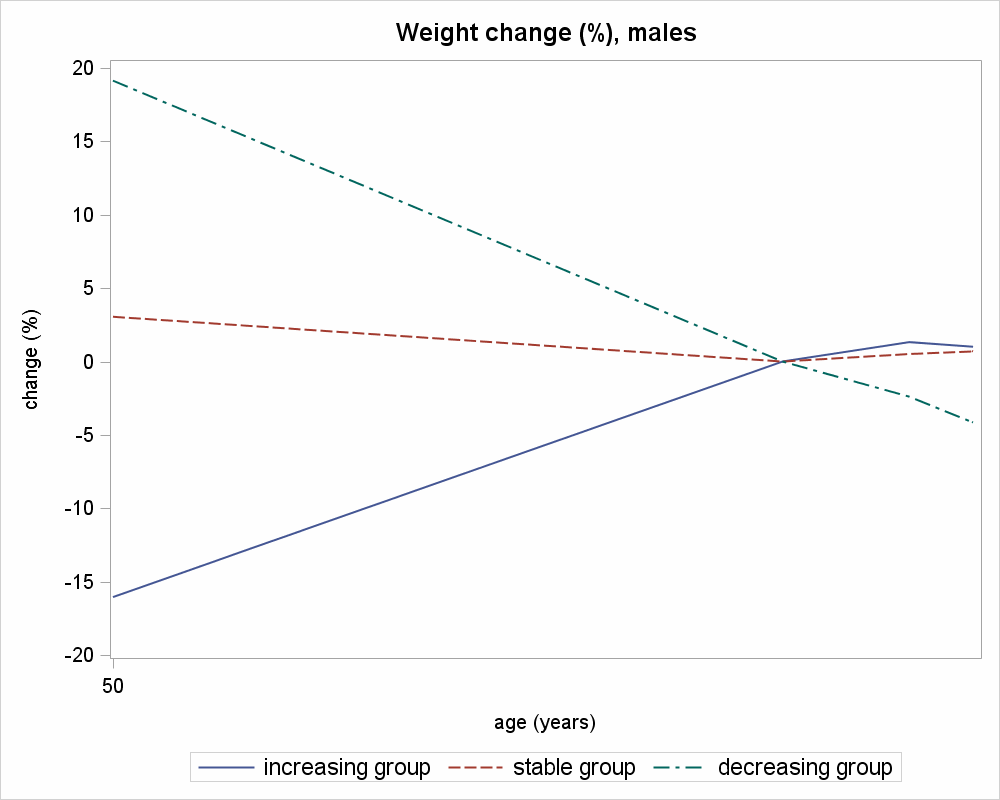


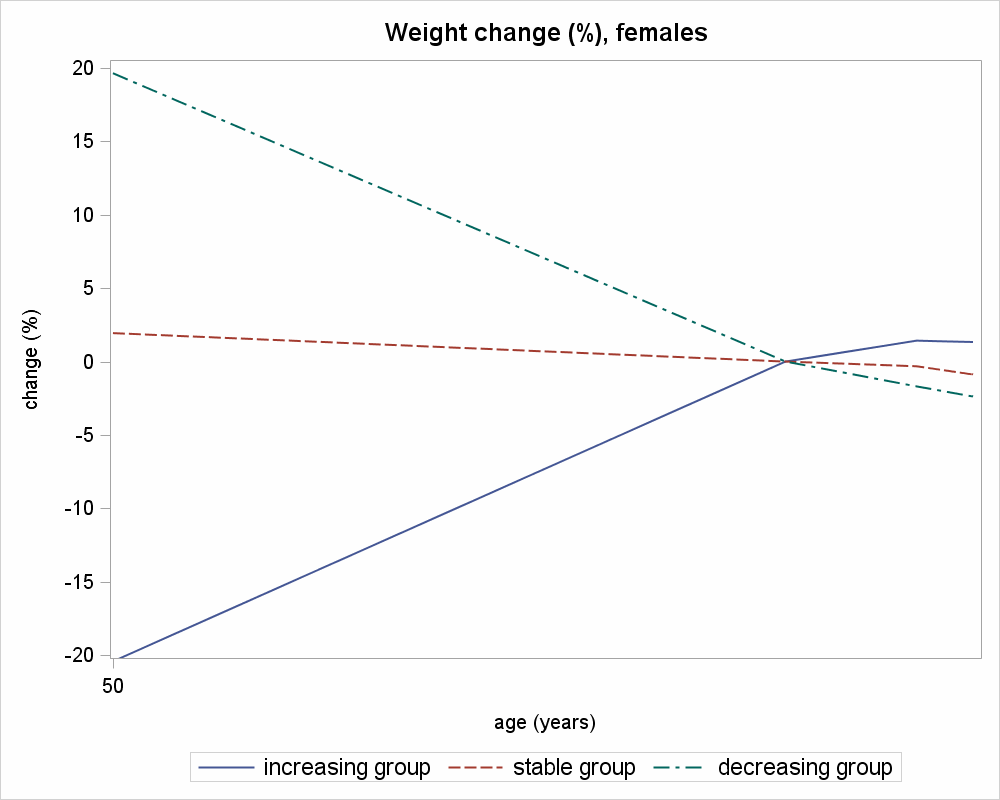


**Supplementary Figure 3.** Weight trajectories identified from baseline to the follow-ups, adjusted for age and sex


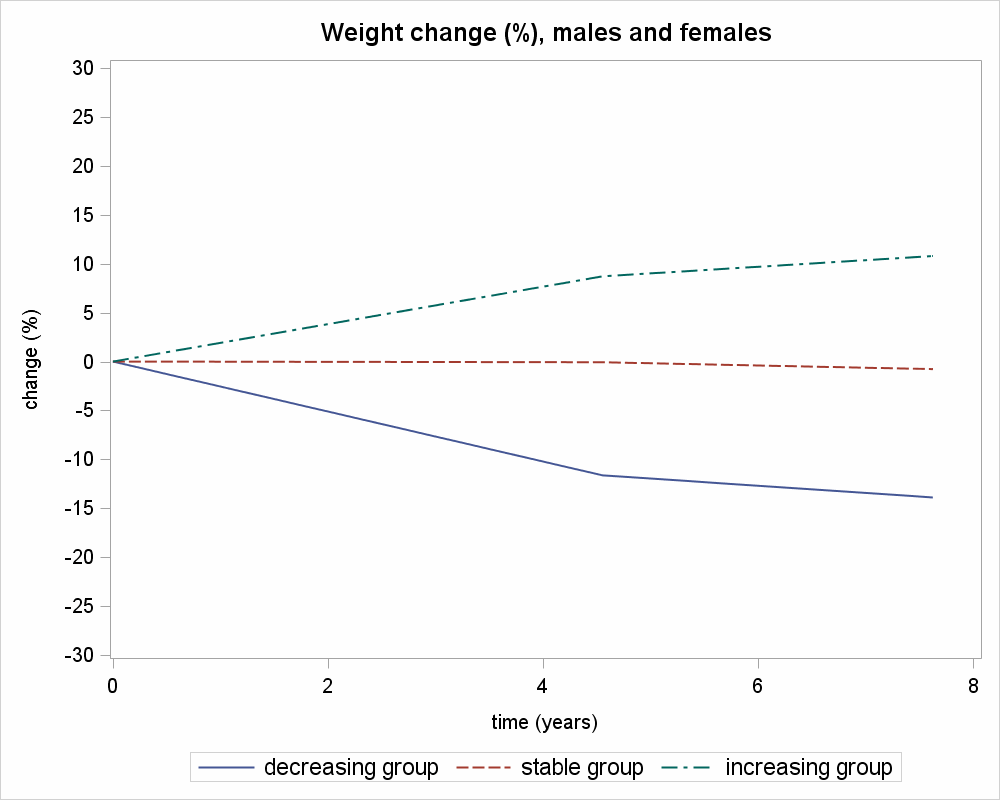


In Cox proportional hazard regression models, taking the stable weight group as the reference, we found no significant interaction between sex and group (p=0.723), and no significant association of the decreasing or increasing trajectories from baseline to follow-up with cognitive decline (HR=0.94, 95%CI: 0.75-1.19, p=0.626 for the increasing weight trajectory; HR=1.19, 95%CI: 0.98-1.46, p=0.085 for the decreasing weight trajectory).

**Supplementary Figure 4.** Weight trajectories identified from age 50 to the follow-up, adjusted for age and sex


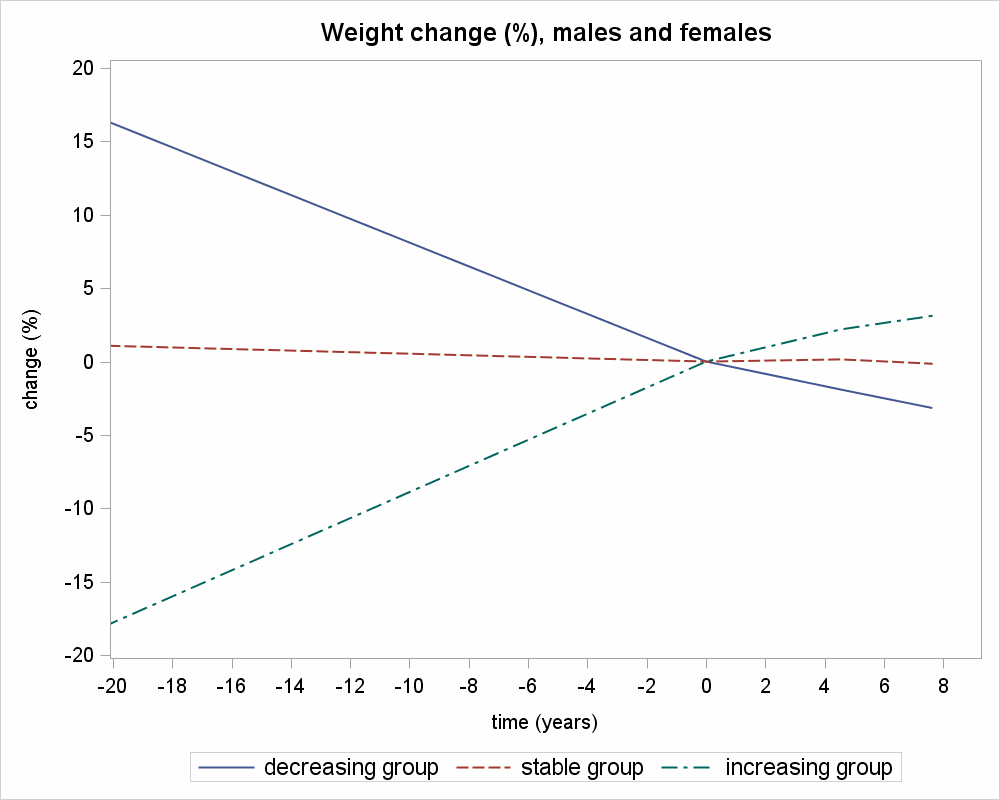


Both the decreasing and the increasing weight trajectory from age 50 were associated with cognitive decline on the MMSE (HR=1.20, 95% CI 1.01-1.41, p=0.034; HR=1.35, 95% CI 1.11-1.64, p=0.003, respectively), adjusting models for sex and age, and the interaction weight trajectory*sex was not statistically significant (p=0.678).
